# Disseminated BCG disease and recurrent pulmonary infections in a child with trisomy 21 carrying a novel *BACH2* variant of uncertain significance: a 5-year case report

**DOI:** 10.3389/fmed.2026.1934925

**Published:** 2026-10-05

**Authors:** Diana Marangu-Boore, Brian Maugo, Rita Afriye Boateng

**Affiliations:** 1 Pediatric Pulmonology Division, Department of Pediatrics and Child Health, University of Nairobi, Nairobi, Kenya; 2 Neonatology Division, Department of Pediatrics and Child Health, University of Nairobi, Nairobi, Kenya; 3 Department of Parasitology, Noguchi Memorial Institute for Medical Research, Accra, Ghana

**Keywords:** *BACH2*, BCG disease, case report, Down syndrome, immunodeficiency, recurrent pneumonia, trisomy 21, variant of uncertain significance

## Abstract

**Background:**

Trisomy 21 is associated with combined immune dysregulation, including lymphopenia, impaired responses to vaccines, and heightened susceptibility to respiratory infections, driven in part by triplication of chromosome 21-encoded interferon receptor genes. *BACH2* haploinsufficiency is a rare monogenic immunodeficiency characterized by lymphopenia, hypogammaglobulinemia, and susceptibility to infections and autoimmune diseases. Co-occurrence of these two conditions has not been previously described.

**Case presentation:**

We report a 5-year longitudinal course in a Kenyan girl with trisomy 21, congenital heart disease, aspiration, and severe gastroesophageal reflux disease (GERD) found via whole exome sequencing to carry a heterozygous *BACH2* missense variant (c.1420C > G;p.Leu474Val) of uncertain significance; parental segregation remains incomplete. In silico protein structural modeling (FoldX and Missense3D) yielded discordant predictions of the variant's effect on the protein’s stability and structure. Additional *in silico* pathogenicity predictions were also inconsistent: Sorting Intolerant from Tolerant (SIFT), Evolutionary Model of Variant Effect (EVE), PolyPhen-2, and CADD Phred scores predicted pathogenicity, while REVEL and AlphaMissense suggested a lack of deleteriousness. Persistent panlymphopenia and profoundly low IgM levels were documented throughout the follow-up period. Over these 5 years, the girl sustained six hospitalized lower respiratory tract infections (LRTIs), with four requiring intensive care, which were disseminated Bacille Calmette-Guérin (BCG) disease, respiratory syncytial virus and SARS-CoV-2 infections, and influenza A(H1N1)pdm09 pneumonia. Chest computed tomography at 4 years showed bilateral consolidations without bronchiectasis. Annual autoimmune screening remained negative.

**Conclusion:**

This case illustrates the burden caused by severe and diverse respiratory pathogenic infections in a child with trisomy 21 and a candidate *BACH2* variant. Trisomy 21 alone plausibly explains much of the phenotype; an independent contribution from the *BACH2* variant remains unproven pending functional and segregation studies. Clinical considerations supported by this case include the potential value of prevaccination immunological screening to prevent BCG disease, equitable access to viral prophylaxis, and early specialist engagement in refractory cases.

## Introduction

Trisomy 21 (Down syndrome) is the most common viable chromosomal aneuploidy. Beyond its neurodevelopmental and structural phenotypes, triplication of interferon receptor genes (*IFNAR1*, *IFNAR2*, *IL10RB*, and *IFNGR2*) encoded by chromosome 21 is associated with constitutive interferon pathway hyperactivation (1, 2), which has been proposed to contribute to panlymphopenia, impaired responses to vaccines, and heightened susceptibility to both infections and autoinflammatory diseases (3–6). Children with trisomy 21 experience disproportionate morbidity from respiratory infections, including viral lower respiratory tract infections (LTTIs) (7, 8).

BACH2 [BTB domain and Cap'n'collar (CNC) homolog 2] is a transcriptional repressor of the BTB-basic leucine zipper (bZIP) family that is highly expressed in B and T lymphocytes, where it represses effector differentiation and sustains generation of regulatory T cells and memory lymphocytes; in B cells, it facilitates germinal center maintenance and immunoglobulin class-switch recombination (9–11). Haploinsufficiency of *BACH2* causes a primary immunodeficiency disorder characterized by lymphopenia, hypogammaglobulinemia, inflammatory bowel disease, and susceptibility to recurrent infections (12), very few cases of which have been described globally, almost exclusively from high-income settings with access to comprehensive genomic platforms (12, 13).

Herein, we report a 5-year prospective case study of a Kenyan girl with trisomy 21 and severe and diverse respiratory diseases, including disseminated Bacille Calmette-Guérin (BCG) disease, congenital heart disease, aspiration, and severe gastroesophageal reflux disease (GERD). Our primary aim is to describe the 5-year clinical and diagnostic course of the child's recurrent and diverse respiratory diseases and the challenges they posed to surveillance and management in a high tuberculosis/BCG-endemic, resource-limited setting. A secondary, exploratory aim is to characterize the possible, unproven contribution of a novel heterozygous *BACH2* missense variant of uncertain significance (VUS; c.1420C > G, p.Leu474Val), also identified in this child. To our knowledge, co-occurrence of trisomy 21 and a candidate *BACH2* variant has not been previously reported. The central interpretive challenge is disentangling immune dysregulation attributable to trisomy 21 itself from any additional contribution of the *BACH2* variant, a distinction that remains unresolved, as parental segregation and functional testing are incomplete. We describe the burden caused by sequential respiratory pathogenic infections, immunological trajectory, and management decisions over the 5-year period, aiming to inform surveillance and management strategies for similarly complex patients in high tuberculosis/BCG-endemic, resource-limited settings.

## Case description

### Integrated clinical timeline

We chronologically summarize the key events across the 5-year course in Figure 1, in addition to providing the respiratory episode detail in Table 1.

**Figure 1:**
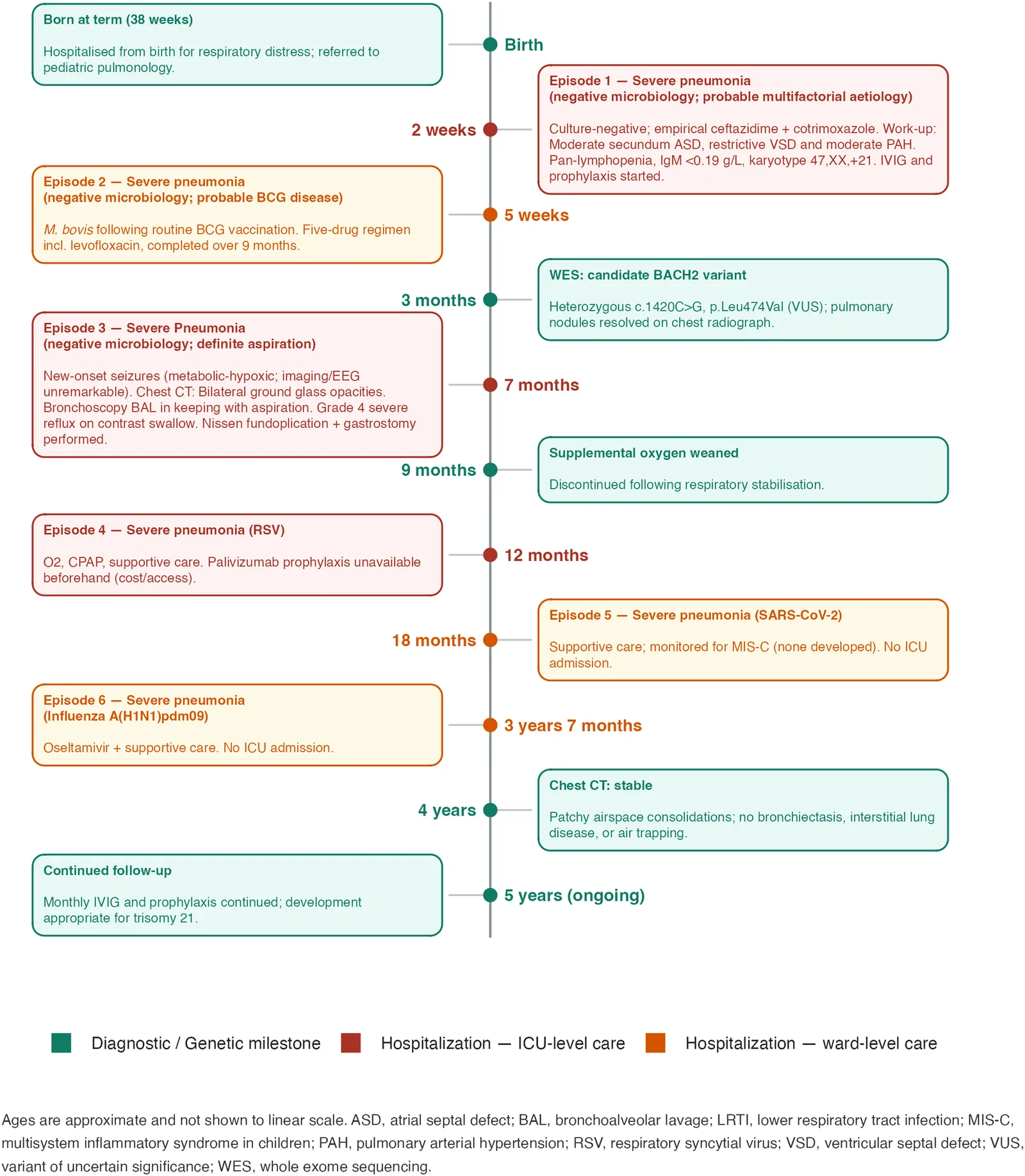
A five-year longitudinal clinical timeline for complex pediatric respiratory illness.

**Table 1:** Summary of immunological findings at presentation.

Parameter	Patient's results (3 weeks: day 18 of life)	Lab reference (0–2 months)
CD3 (cells/μL)	1,233	2,500–5,500^a^
CD4 (cells/μL)	622	1,600–4,000^a^
CD8 (cells/μL)	335	560–1,700^a^
CD19 (cells/μL)	133	300–2,000^a^
CD56 (cells/μL)	106	170–1,100^a^
IgG (g/L)	5.56	5.04–14.64
IgM (g/L)	<0.188	0.24–2.10
IgA (g/L)	<0.279	0.27–1.95
IgE (g/L)	ND	NA

ND, not done; NA, not applicable.

a Lab reference values based on Shearer et al. (14).

### Clinical presentation

The patient is a female infant with dysmorphic features who was born at term (38 weeks) by caesarean section to non-consanguineous Kenyan parents. She was hospitalized from birth for respiratory distress and referred to the pediatric pulmonology department at the age of 2 weeks. Key comorbidities at referral were a moderate secundum atrial septal defect (ASD), restrictive ventricular septal defect (VSD), and moderate pulmonary arterial hypertension (PAH) revealed by echocardiography and persistent airspace consolidations revealed by plain chest radiography. The cardiac conditions were managed by a combinatorial treatment comprising digoxin, furosemide, aldactone and sildenafil, and they were monitored via serial echocardiography, with these conditions improving in the first 4 weeks from diagnosis, thereby requiring no surgical closure. Medical management of aspiration and gastroesophageal reflux disease was instituted on clinical grounds, specifically recurrent postfeed desaturations and vomiting, despite an initial contrast swallow test result that was negative for aspiration but showed grade 2 reflux.

Further investigations revealed panlymphopenia with each subset below laboratory reference ranges [CD3^+^, 1,233 cells/μL (2,500–5,500); CD4^+^, 622 cells/μL (1,600–4,000); CD8^+^, 335 cells/μL (560–1,700); CD19^+^, 133 cells/μL (300–2,000); CD16/56^+^, 106 cells/μL (170–1,100)]; the lower range of normal serum IgG levels [5.56 g/L (5.04–14.64)] reflective of maternal IgG transferred via the placenta, and profoundly low serum IgM levels (below the assay’s lower limit of detection, <0.188 g/L). Trisomy 21 was confirmed on the karyotype (47, XX, +21). The FISH test result for 22q11.2 deletion was negative. Their family history was notable for recurrent infections and autoimmune disease in a first-degree relative, raising the possibility of a familial predisposition to immunodeficiency and/or autoimmunity. Intravenous immunoglobulin (IVIG) therapy was initiated at the age of 3 weeks, once the immunological results became available.

### Genetic findings and protein structural analysis

At the age of 3 months, whole exome sequencing (WES) using a commercial platform identified a heterozygous missense variant in *BACH2* (NM_021813.4): c.1420C > G (p.Leu474Val), which was classified as a variant of uncertain significance in accordance with the American College of Medical Genetics criteria and was absent from gnomAD (v3.1.2). Parental segregation testing is incomplete, and therefore, *de novo* occurrence cannot be confirmed yet.

The human *BACH2* gene encodes a large, ∼130 kDa transcriptional repressor comprising a Broad-complex, Tramtrack, and Bric-à-brac (BTB/POZ) domain, a bZIP domain responsible for DNA binding, and a CNC domain (15). BACH2 represses gene transcription by forming heterodimers with small Maf proteins via the bZIP domain, regulating immune cell differentiation and maintaining lymphocyte homeostasis (16), and is an emerging target for immune-modulating therapies (17).

The variant p.Leu474Val is situated within an intrinsically disordered region (IDR; residues 331–520) lying between the BTB and bZip domains. This region contains three of the five cysteine–proline (CP) motifs implicated in heme binding. Heme binding to these motifs has been shown to inhibit BACH2 DNA-binding activity and reduce protein stability. FoldX modeling predicted a negligible, marginally stabilizing effect of the Leu474Val substitution on global protein stability (ΔΔG ≈ −0.01 kcal/mol).

An independent structural impact assessment using Missense3D (https://missense3d.bc.ic.ac.uk/) predicted that the substitution at position 474 causes a significant structural disturbance. These two computational predictions are discordant: FoldX predicted minimal impact on global thermodynamic stability, while Missense3D predicted substantial local structural disruption.

The pathogenicity of the BACH2 p.Leu474Val missense variant was predicted using three independent computational prediction tools: Sorting Intolerant from Tolerant (SIFT) (18), the Evolutionary Model of Variant Effect (EVE) (19), and PolyPhen-2 (20). All these tools predicted a potentially pathogenic effect of the variant, although their scoring systems and classification criteria differed. SIFT assigned a score of 0.00 and classified the variant as deleterious, indicating a high predicted likelihood of functional intolerance to amino acid substitution. EVE assigned a score of 0.690 and classified the variant as pathogenic based on the evolutionary constraint. PolyPhen-2 assigned a score of 0.996 and classified the variant as probably damaging, providing strong evidence for a potential adverse effect on protein function. Collectively, the concordant predictions from SIFT, EVE, and PolyPhen-2 support the potential pathogenicity of the BACH2 p.Leu474Val variant.

Additional in silico pathogenicity predictors were also examined using MobiDetails (https://mobidetails.chu-montpellier.fr). The CADD Phred score for this variant is 24.2, indicating moderate-to-high predicted deleteriousness relative to other genome-wide variants. The REVEL prediction score is 0.21, which is classified as uncertain, whereas the AlphaMissense prediction score is 0.084, which is classified as likely benign, mirroring the discordance observed with structural modeling. As with the FoldX, Missense3D, SIFT, PolyPhen-2, and EVE results, these scores are presented as hypothesis-generating and do not, individually or in combination, establish the pathogenicity of this VUS.

Taken together with the variant's location within a functionally important heme-sensing motif, these findings are hypothesis-generating rather than conclusive; functional lymphocyte studies and completion of parental and relative segregation testing have been planned, which can determine the variant's true impact on BACH2 activity and formally reclassify the variant (Figure 2 and Table 2).

**Figure 2:**
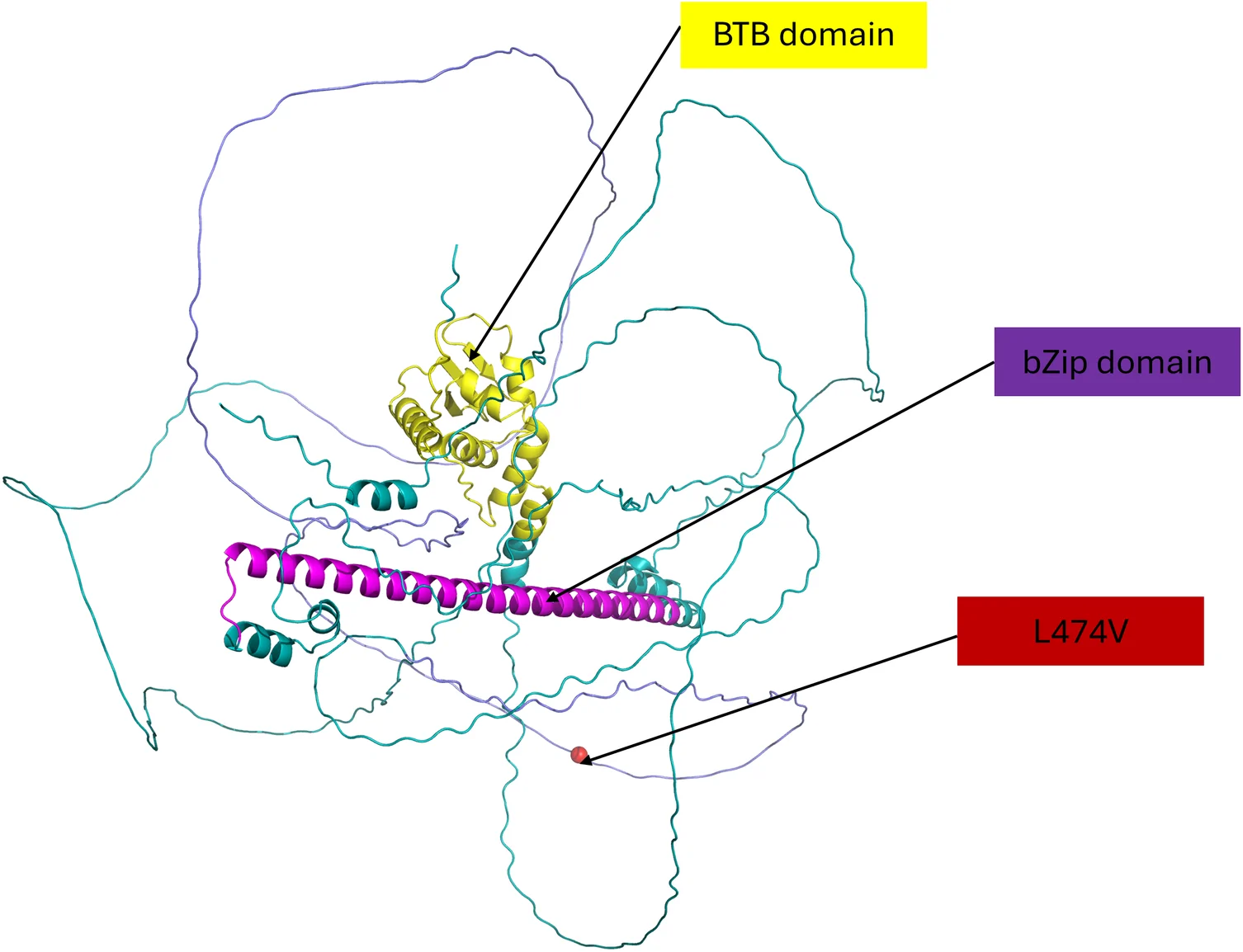
The position of the p.Leu474Val variant on the BACH2 protein structure (red sphere). The BTB domain is shown in yellow, and the bZIP domain is shown in magenta.

**Table 2:** In silico pathogenicity prediction scores for the *BACH2* p.Leu474Val variant.

Pathogenicity Prediction Tool	Score	Prediction
Sorting intolerance from tolerance (SIFT)	0.00	Deleterious
Evolutionary model of variant effect (EVE)	0.690	Pathogenic
PolyPhen-2	0.996	Probably damaging
CADD Phred score	24.2	Moderate-to-high deleteriousness
REVEL	0.21	Uncertain
AlphaMissense	0.084	Likely benign

A copy number variation (CNV) analysis of exome data identified an apparent duplication within chromosome 21 against a constitutional trisomy 21. Diploid-referenced CNV calling is unreliable in this setting, and this finding most likely represents an algorithmic artifact of trisomy 21 rather than a true additional copy number gain; it is reported for completeness rather than as an established clinical finding. The candidate duplicated region includes IFNGR2; in trisomy 21, IFNGR2 overexpression has been proposed to produce interferon pathway hypersensitivity (a gain-of-signal mechanism), which is mechanistically distinct from Mendelian susceptibility to mycobacterial disease, a condition caused by a biallelic IFNGR2 loss-of-function mutation.

In addition, heterozygous variants were identified in *DNAH11* (c.6244C > T; p.Arg2082*, pathogenic) and *CFTR* (c.120-34TG[11]T[5], an intronic, low-penetrance allele), consistent with the carrier status of primary ciliary dyskinesia and a low-penetrance CFTR-related risk, respectively. Sweat conductivity was 44 mmol/L NaCl equivalent (normal, <50 mmol/L).

### Immunological profile

Serial immunological phenotyping over 5 years consistently demonstrated panlymphopenia with persistently reduced T, B, and NK cell subsets. CD4^+^ T-cell counts remained below the lower limit of even the trisomy 21-adjusted reference ranges throughout the follow-up. IgM levels remained below the assay's lower limit of detection. Following initiation of monthly IVIG at the age of 3 weeks (0.4 g/kg), IgG trough levels ranged from 510 to 740 mg/L, consistently above the 500 mg/L minimum replacement target. IgA levels were persistently low. Annual autoimmune screening panels (antinuclear antibody and anti-double-stranded DNA) were negative throughout the follow-up (Table 3, Figures 3, 4).

**Table 3:** Lymphocyte subsets reference values in children who are healthy or have trisomy 21 by age in the first 5 years.

Lymphocyte subset (cells/μL)	Healthy reference^a^ (0–2 months)	Healthy reference^a^ (3–5 months)	Healthy reference^a^ (6–11 months)	Healthy reference^a^ (12–23 months)	Trisomy 21 reference^b^ (6–23 months)	Healthy reference^a^ (2–5 years)	Trisomy 21 reference^b^ (2–6 years)
CD3	2,500–5,500	2,500–5,600	1,900–5,900	2,100–6,200	1,450.80 ± 324.49	1,400–3,700	1,161.47 ± 374.25
CD4	1,600–4,000	1,800–4,000	1,400–4,300	1,300–3,400	668.00 ± 139.46	700–2,200	500.93 ± 164.84
CD8	560–1,700	590–1,600	500–1,700	620–2,000	782.80 ± 226.23	490–1,300	657.87 ± 236.00
CD19	300–2,000	430–3,000	610–2,600	720–2,600	416.40 ± 126.09	370–1,400	412.13 ± 116.15
CD56/16	170–1,100	170–830	160–950	180–920	185.60 ± 076.33	130–720	230.67 ± 091.18

a Lab reference values based on Shearer et al. (14).

b Egyptian study by Mitwalli et al. (21).

**Figure 3:**
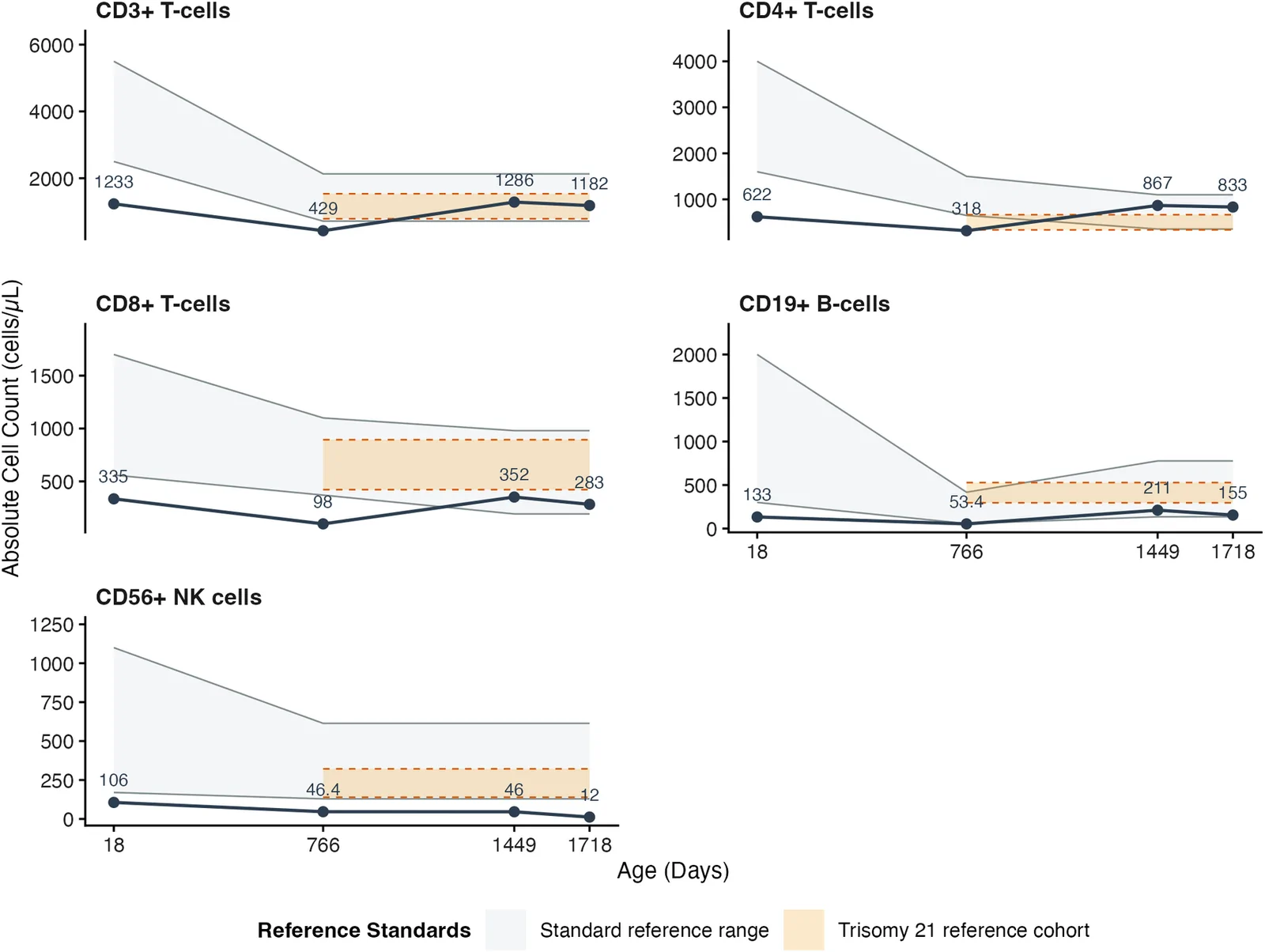
Absolute CD3^+^ T cell, CD4^+^ T cell, CD8^+^ T cell, CD19^+^ B cell, and CD56/16 NK cell counts mapped against age-specific reference bounds over the 5-year follow-up.

**Figure 4:**
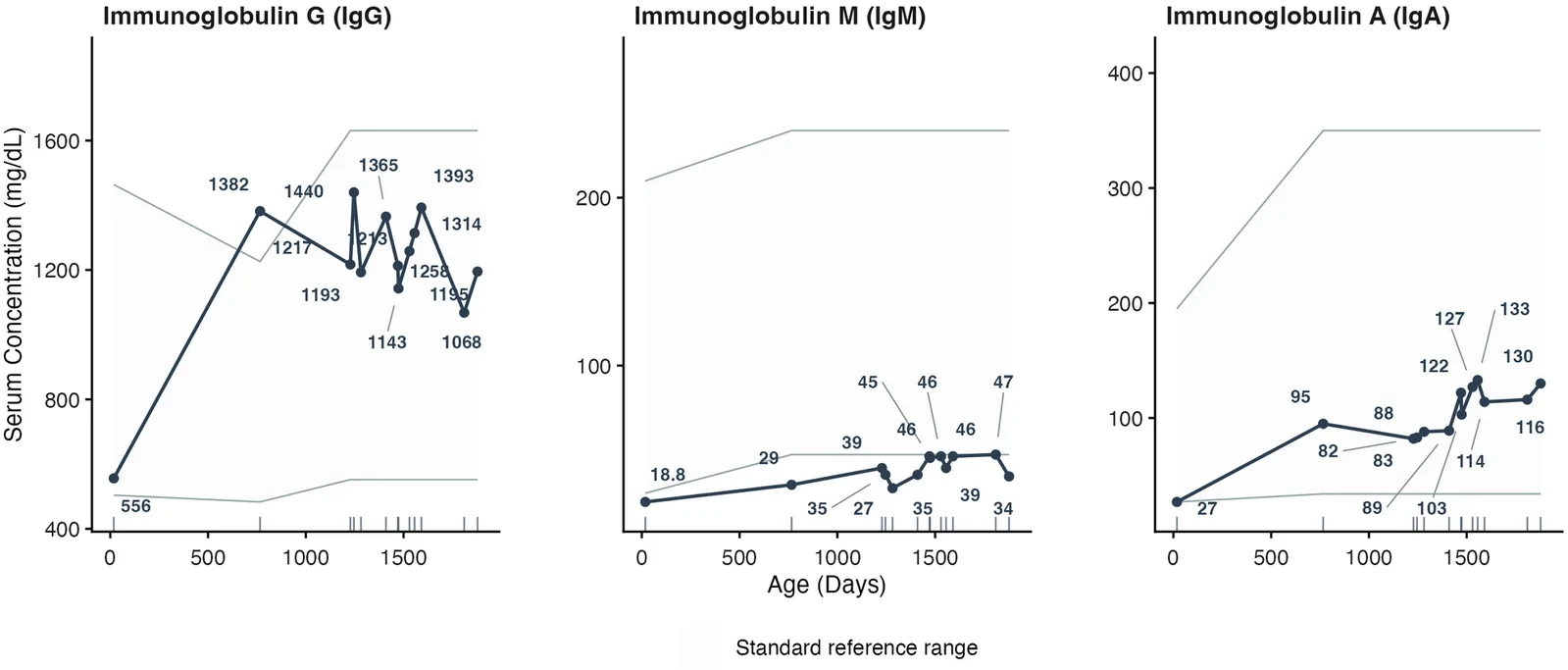
Quantitative serum immunoglobulin G (pre-IVIG at baseline, subsequent trough levels), immunoglobulin M, and immunoglobulin A levels mapped against age-specific reference bounds over the 5-year follow-up.

## Diagnostic assessment, intervention, follow-up, and outcomes

All significant LRTIs for which the child was hospitalized are summarized in Table 4.

**Table 4:** Summary of hospitalized lower respiratory tract infection episodes, 2020–2026.

Episode	Age	Presentation/pathogen	ICU	Management	Outcome	Diagnostic certainty of a microbiological etiology
1	2 weeks	Neonatal sepsis/severe pneumonia. Bilateral opacities; respiratory distress; no pathogen identified (culture-negative) CMV PCR negative (lower limit of detection, 100 copies/mL)	Yes	Empirical ceftazidime + co-trimoxazole; IVIG commenced; prophylaxis initiated	Improved	Uncertain—Possible: Culture-negative (empirical diagnosis; no pathogen identified)
2	5 weeks	Severe pneumonia. Disseminated BCG disease (*M. bovis*); TST+; Xpert MTB/RIF negative on induced sputum; 1 week post-BCG vaccination	Yes	Isoniazid + rifampicin + pyrazinamide + ethambutol + levofloxacin×9 months	Improved	Uncertain—Probable: Clinically diagnosed (TST-positive, compatible radiographic evolution); not microbiologically confirmed, Xpert MTB/RIF negative, a recognized assay limitation for *M. bovis*
3	7 months	Severe pneumonia (probable aspiration) with new-onset generalized seizures; cultures/PCR negative; brain CT/EEG unremarkable; contrast swallow showed severe grade 4 reflux; bronchoscopy and BAL were consistent with aspiration; HRCT chest showed bilateral ground glass opacities	Yes	Empirical antibiotics; anticonvulsants; Nissen fundoplication + gastrostomy	Improved; O_2_ weaned at 9 months	Uncertain—Possible: Clinical diagnosis of aspiration; cultures/PCR negative
4	12 months	Severe RSV pneumonia (BioFire NPA positive)	Yes	O2, CPAP, supportive care; palivizumab not obtained (cost/availability)	Improved	Certain: Microbiologically confirmed (BioFire NPA positive)
5	18 months	Severe SARS-CoV-2 pneumonia (NPA PCR positive)	No	O2, supportive; monitored for MIS-C (none developed)	Improved	Certain: Microbiologically confirmed (BioFire NPA positive)
6	3.6 years	Severe influenza A(H1N1)pdm09 pneumonia (NPA PCR positive)	No	O2; oseltamivir; supportive	Improved	Certain: Microbiologically confirmed (BioFire NPA positive)

BCG, Bacille Calmette-Guérin; CMV, cytomegalovirus; CPAP, continuous positive airway pressure; ICU, intensive care unit; IVIG, intravenous immunoglobulin; MIS-C, multisystem inflammatory syndrome in children; NPA, nasopharyngeal aspirate; PCR, polymerase chain reaction; RSV, respiratory syncytial virus; TST, tuberculin skin test.

The earliest LRTI episode, at the age of 2 weeks, was a culture-negative bilateral pneumonia treated empirically with broad-spectrum antibiotics and supportive respiratory care, alongside initiation of monthly IVIG and a prophylactic regimen (co-trimoxazole, azithromycin, acyclovir, and fluconazole). The result of a cytomegalovirus (CMV) polymerase chain reaction (PCR) test of the blood was negative (lower limit of detection, 100 copies/mL).

One week after discharge, at 5 weeks, the patient was readmitted with disseminated BCG disease following routine BCG vaccination given in accordance with Kenya's national immunization schedule. Diagnosis was established clinically (positive tuberculin skin test and progressive radiographic changes) rather than microbiologically; the GeneXpert MTB/RIF assay on induced sputum produced negative results. Treatment required a five-drug regimen including levofloxacin, reflecting the intrinsic pyrazinamide resistance of *Mycobacterium bovis*, which was completed in 9 months. Resolution of pulmonary nodules was evident on chest radiography following treatment for disseminated BCG disease at the age of 3 months (Figure 5).

**Figure 5:**
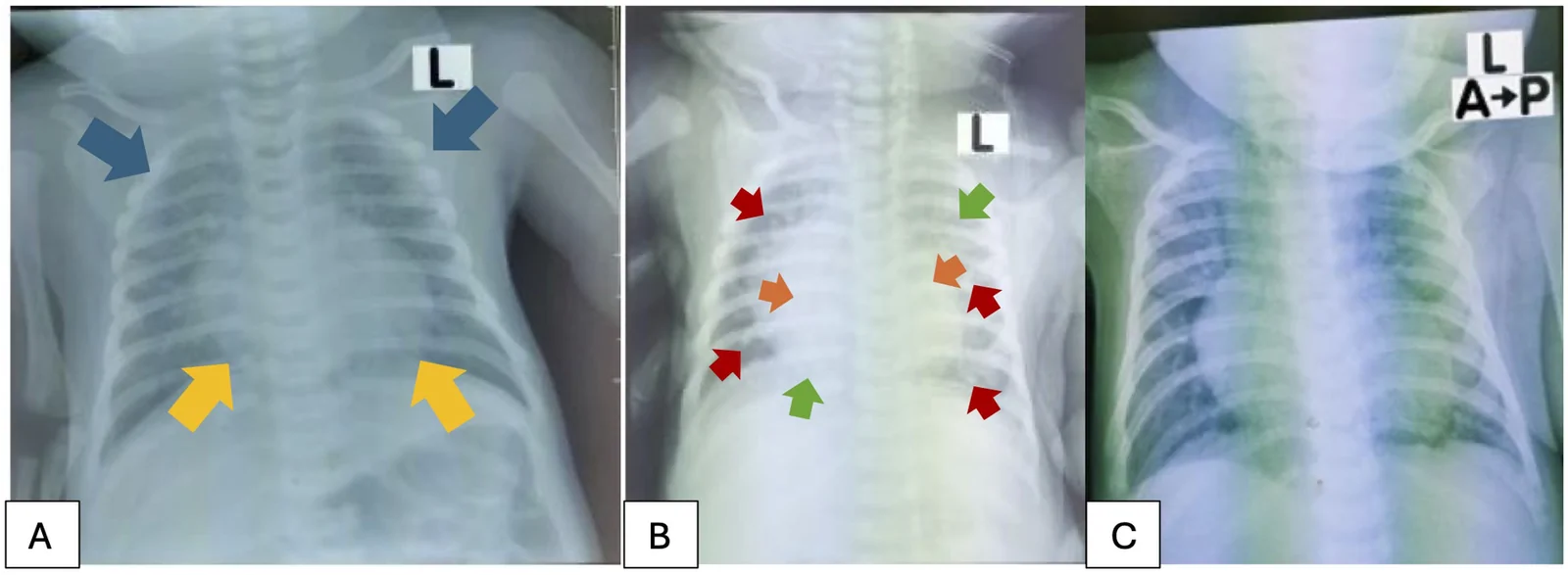
Serial chest radiographs in infancy. **(A)** At 2 weeks: diffuse ground glass opacification (blue arrows) and cardiomegaly (yellow arrows). **(B)** At 5 weeks: diffuse nodular (red arrows) and patchy airspace opacities (green arrows) with more prominent air bronchograms (orange arrows) in the right and left upper lobes (rotated image) suggestive of interval worsening and concurrent with disseminated BCG disease. **(C)** At 3 months: resolution of pulmonary nodules following treatment for disseminated BCG disease.

At the age of 7 months, severe pneumonia complicated by new-onset seizures (attributed to acute metabolic-hypoxic insult; brain imaging and EEG were unremarkable) prompted a multidisciplinary review. A repeat modified contrast swallow study confirmed severe GERD and aspiration, for which Nissen fundoplication and gastrostomy were performed, respectively. Supplemental oxygen was subsequently weaned by the age of 9 months.

Severe pneumonia caused by respiratory syncytial virus (RSV) infection at the age of 12 months required ICU-level respiratory support. Palivizumab prophylaxis had not been administered beforehand because of local cost and availability constraints, and a later compassionate use was declined, an access gap the family has continued to grapple with for subsequent RSV seasons. Severe pneumonia caused by SARS-CoV-2 infection at the age of 18 months and that by influenza A(H1N1)pdm09 at the age of 3 years and 7 months were each managed successfully with supportive care and, for influenza, oseltamivir, without ICU admission.

Chest CT at the age of 4 years showed bilateral patchy airspace consolidations consistent with the patient’s infectious history, without bronchiectasis, interstitial lung disease, air trapping, or nodules, a reassuring structural baseline. She continues outpatient follow-up with pulmonology, immunology, and cardiology teams, remains on her prophylactic regimen (co-trimoxazole, azithromycin, acyclovir, and fluconazole) and monthly IVIG (dosed to maintain IgG troughs above the 500 mg/L replacement target), and has required no supplemental oxygen since the weaning at the age of 9 months apart from the acute hospitalizations. Hospitalizations have become markedly less frequent than in the first 2 years of life, and she has shown sustained growth (her current weight is 21 kg—trisomy-adjusted 50th percentile, her current height is 105 cm—trisomy-adjusted above the 95th percentile, and her current body mass index is 19.1, above the trisomy-adjusted 95th percentile). She meets developmental milestones appropriate for trisomy 21, with ongoing physiotherapy and early intervention support. Immunologically, she continues to show persistently low lymphocyte subsets (predominantly NK cells), IgM levels below the assay's lower limit of detection, and IgG troughs maintained above the replacement target on IVIG. Functional lymphocyte studies (including flow cytometric assessment of regulatory cell, Th1, naïve T cell, and class-switched memory B-cell frequencies, hallmarks reported in BACH2 haploinsufficiency), BACH2 protein expression assays by flow cytometry/immunoblotting, and completion of parental and first-degree relative segregation testing, prioritizing the relative with recurrent infections and autoimmune disease, have been planned to further characterize the variant and refine its classification.

## Discussion

### Interpretation of the BACH2 variant and trisomy 21 as an immunological modifier

This 5-year case illustrates convergent immune vulnerability in a child with trisomy 21 and a candidate *BACH2* variant, a combination not previously reported. Trisomy 21-driven interferon pathway hyperactivation, reduced lymphocyte counts, and impaired antibody responses plausibly account for much of this patient's phenotype. Whether the *BACH2* variant contributes independently remains unresolved. Its location within a heme-binding regulatory motif is biologically possible but not established. FoldX and Missense3D yielded discordant structural predictions. Additional in silico predictions were similarly inconsistent (SIFT, EVE, PolyPhen-2, and CADD Phred scores predicted pathogenicity, whereas REVEL and AlphaMissense suggest a lack of deleteriousness). Parental segregation testing is incomplete, and therefore, neither *de novo* status nor co-segregation with disease can be confirmed yet. We deliberately avoid overstating the variant's pathogenicity. Functional lymphocyte and protein expression studies, together with the completion of parental testing, are required before this VUS can be reclassified.

An additional avenue worth noting, purely for completeness, is the recognized role of PI3 K/mTORC1 signaling in promoting phosphorylation and the nuclear export of BACH2, thereby attenuating its transcriptional repressor activity (22). Should future functional studies confirm that p.Leu474Val compromises BACH2 activity, modulation of this signaling axis, for example, with the PI3Kδ inhibitor leniolisib or mTOR inhibitors such as rapamycin, both used in other PI3K-driven immune dysregulation syndromes, represents a biologically possible, although presently speculative, therapeutic avenue distinct from trisomy-21-related mechanisms. This possibility is not a treatment recommendation for this patient currently.

### Disseminated BCG disease as a sentinel finding

Disseminated BCG disease in this infant underscores a persistent vaccine-safety challenge in countries with a universal BCG policy. Live attenuated BCG is contraindicated in primary immunodeficiency, yet the immunological status is rarely established before the routine neonatal schedule in resource-limited settings. In this case, the immunological workup had already revealed combined T and B immunodeficiency and BCG vaccination had accordingly been documented as withheld. It was nonetheless inadvertently administered as a routine vaccine at discharge, a failure of the system illustrating how easily such holds are missed in busy neonatal units without reliable safeguards. The World Health Organization recommends deferring BCG pending immunological evaluation in infants with suspected T-cell deficiency, but systematic prevaccination screening remains logistically difficult where laboratory capacity is limited (23, 24). The negative Xpert MTB/RIF result despite confirmed BCG disease is a clinically important caveat. This assay has a recognized limitation in detecting BCG (*M. bovis*) strains and should not be relied upon to exclude BCG disease in an immunocompromised host (25).

### Surveillance, prophylaxis, and equity

The spectrum of pathogens in this patient—bacterial, mycobacterial, and viral—reflects the wide-ranging prophylaxis needs of severe combined immune dysregulation (26). Gaps in access to RSV immunoprophylaxis (palivizumab; now longer-acting agents such as nirsevimab) and to comprehensive microbiological workup at every episode reflect a recurring inequity in global pediatric immunology practice that limited clinical care for this patient (27). Persistent panlymphopenia and recurrent severe LRTIs despite maximal medical prophylaxis raise the question of curative intervention. To date, there are no documented cases of hematopoietic stem cell transplantation reconstituting immune function specifically in *BACH2* haploinsufficiency, although it remains a biologically possible option given the outcomes in other combined T- and B-cell immunodeficiencies with overlapping phenotypes (28). For this patient, any such consideration would need to account for concurrent trisomy 21 and residual pulmonary disease.

This report carries the inherent limitations of case reports: the findings are not generalizable, and causal inference cannot be drawn. Several specific limitations warrant emphasis. First, the *BACH2* variant remains functionally uncharacterized. Comprehensive lymphocyte phenotyping (T regulatory cell, Th1, naïve T cell, and class-switched memory B-cell frequencies), BACH2 protein expression measurement by flow cytometry/immunoblotting, and functional studies have not yet been performed. Parental and first-degree relative segregation testing, the single most informative next step for variant classification, was not available at the time of this report but is being actively pursued. Second, disseminated BCG disease was diagnosed clinically rather than microbiologically, as the GeneXpert MTB/RIF assay on induced sputum was negative, a recognized limitation of this assay for *M. bovis*. Third, congenital heart disease, aspiration, and severe GERD are major well-documented non-immunological contributors to this patient's respiratory morbidity that cannot be fully disentangled from any immunodeficiency-related contribution. Fourth, autoimmune surveillance was limited to antinuclear antibody and anti-double-stranded DNA testing and may not capture the full autoimmune spectrum associated with BACH2 haploinsufficiency. Finally, resource constraints precluded palivizumab prophylaxis, and detailed flow cytometric phenotyping for lymphocyte subsets reported in BACH2 haploinsufficiency has not yet been performed.

## Conclusion

This case documents a severe and diverse burden by respiratory pathogenic infections, including disseminated BCG disease, in a child with trisomy 21 and a candidate *BACH2* variant of uncertain significance. It raises considerations that are illustrative rather than generalizable recommendations from a single case, regarding the potential value of prevaccination immunological screening, equitable access to viral prophylaxis, and early specialist engagement in refractory pediatric immunodeficiency, particularly in high-burden, resource-limited settings.

## Patient perspective

It has not been an easy journey for our daughter over the years, but as a family, we have witnessed genuine progress. She is steadily meeting several of her developmental milestones and has gained weight, which we regard as a meaningful sign of improvement. Emergency hospital visits, once frequent in her early years, have also become far less common.

Despite this progress, we continue to hope for a permanent solution to her condition—one that would allow her to engage fully and freely with her peers without the constant fear of severe infection.

## Data Availability

The original contributions presented in the study are included in the article, further inquiries can be directed to the corresponding author/s.
